# Identification of Different Profiles of Illness Perception in COPD Patients: Results of Cluster Analysis

**DOI:** 10.2174/18743064-v16-e2112141

**Published:** 2022-02-08

**Authors:** Svetlana Ovcharenko, Yanina Galetskayte, Dmitry Romanov, Dmitry Petelin, Beatrice Volel

**Affiliations:** 1 Department of Faculty Therapy, Sechenov University, Moscow, Russian Federation; 2 Department of Psychiatry and Psychosomatics, Sechenov University, Moscow, Russian Federation; 3 Mental Health Research Center, Moscow, Russian Federation

**Keywords:** COPD, Illness perception, Anxiety, Depression, Smoking, Dyspnea

## Abstract

**Background:**

Chronic obstructive pulmonary disease (COPD) is a widespread, late-diagnosed, and difficult-to-treat disease that influences the quality of life. Despite the availability of a wide range of drugs for the treatment of COPD, none of them provides a complete cure, while the leading risk factors (primarily, smoking) persist. In this regard, illness perception and medical behavior play a key role.

**Methods:**

The study design was cross-sectional and included 143 stable outpatients (107 men, mean age 66 ± 7.5, FEV1 51.5 ± 16.5%) who attended the faculty therapy clinic of Sechenov University. The patients were examined pulmonologically and psychiatrically (Hamilton depression and anxiety rating scales). Illness perception was assessed by a brief version of the Illness perception questionnaire (brief IPQ).

**Results:**

There were no significant demographic differences and differences in the clinical severity of the disease between the selected groups. Patients in the distressed group had a longer duration of illness, a higher prevalence of anxiety and depression, and more severe dyspnea after a 6-minute walk test. In contrast, patients in the disregarding group had a significantly higher prevalence of smokers and a higher number of cigarettes smoked daily, and a lower prevalence of anxiety and depression. The harmonic had the most optimal profile with low severity of anxiety and depression, but with a healthier attitude to smoking.

**Conclusion:**

Perception of illness in COPD patients has a significant impact on medical behavior and levels of anxiety and depression. As such, the perception of illness deserves routine monitoring in clinical practice.

## 1 INTRODUCTION

Chronic obstructive pulmonary disease (COPD) is a widespread, late-diagnosed, and difficult-to-treat disease that influences the quality of life [1-3]. According to the results of the international BOLD study, COPD of stages II and higher is diagnosed in 10.1% of the population over the age of 40. COPD has been found in every fifth long-term smoking patient seeking medical care [4]. In the Russian Federation, COPD pervalence is estimated at around 7%, which corresponds to 11 million patients [5].

Despite the availability of a wide range of drugs for the treatment of COPD, none of them provides a complete cure, especially while the leading risk factors persist, primarily smoking [6]. In this regard, a patient’s awareness of the nature of their disease and their reaction to it are of great importance.

Currently, more attention is paid to how patients respond to chronic somatic disease, which is considered in the framework of the methodology of “illness perception” [7, 8]. Illness perception has a significant impact on medical behavior, adherence to treatment recommendations, and lifestyle adjustments [9]. The analysis of relevant literature reveals that patients demonstrate an ambiguous perception of COPD. On the one hand, anxiety and depression are widespread in such patients, reaching 96% at the terminal stage [10-13]. On the other hand, among patients with COPD, there are people with immature psychological defenses, such as denial or undoing [14].

It can be assumed that abnormal behavior of any type can have a negative impact on the prognosis of the disease. The predominance of catastrophization, hypertrophied perception, and significant underestimation of the severity of this disease are associated with low adherence to therapy and disregard for recommendations to quit smoking. Therefore, the profiles of the COPD perception deserve additional clarification.

Earlier, when examining a smaller cohort of patients using the phenomenological method, we identified four types of illness perception, 1) harmonious, 2) anxious, 3) depressed, and 4) a type inclined to deny their disease [15]. Considering that the phenomenological method is highly subjective, it was decided to test if it is possible to identify these types of illness perception using validated questionnaires.

### 1.1 Aim of the Study

To identify types of illness perception in stable outpatients with COPD using cluster analysis of the Brief IPQ.

## 2 MATERIALS AND METHODS

The study was performed at the Department of Internal Medicine No. 1 and the Department of Psychiatry and Psychosomatics of the Faculty of Medicine of V.N. Vinogradov Faculty Therapeutic Clinic of I.M., Sechenov First Moscow State Medical University (Sechenov University).

All patients signed a voluntary informed consent form to participate in the study. The study was approved by the local ethics committee of Sechenov University.

The following inclusion criteria were applied: the patients were 40 to 80 years old; they had a confirmed mild, moderate, severe, and very severe COPD diagnosis [16]; they had emphysematous, bronchial, or mixed disease types; the duration of the disease was longer than 2 years; COPD exacerbations were absent in the three months preceding this study.

The following exclusion criteria were applied: decompensation of concomitant diseases and the manifestation of respiratory failure, interfering with the patient's questioning; the presence of malignancies at the time of examination; the presence of distinct mental disorders (schizophrenia, bipolar disorder, organic CNS diseases, alcohol and substance abuse).

### 2.1 Somatic Medical Examination

The following somatic medical examinations were conducted for all patients:

● Collection and analysis of complaints, with the assessment of the severity of dyspnoea, cough and sputum production, using the COPD assessment test (CAT) and the Modified British Medical Research Council (mMRC) test; analysis of the patient's disease and life history;

● Collection and analysis of socio-demographic data (education, social status, degree of disability), smoking status (attempts to quit smoking, smoking duration, number of cigarettes per day, and smoking index − SI), data on the patient's history of occupational hazards, frequent hypothermia and catarrhal diseases, and hereditary burden of lung diseases;

● Standard laboratory (general clinical, biochemical and immunological blood tests, general clinical and bacteriological examination of sputum) and instrumental studies (electrocardiography, echocardiography, radiography or computed tomography of chest organs).

### 2.2 Functional Examination

The following functional tests were conducted for all patients.

*Pulmonary function examination*: A portable Schiller SP-1 device (Switzerland) was used for spirometry. The study was conducted according to a generally accepted method. The following parameters were analyzed: forced inspiratory vital capacity (FVC); forced expiratory volume in 1 second (FEV1); forced expiratory flow at 25% FVC (FEF25), 50% FVC (FEF50), and 75% FVC (FEF75), expressed in percent of the expected values; the modified Tiffeneau index (FEV1/FVC ratio). All patients were also tested for the reversibility of bronchial obstruction with a bronchodilator (inhalation of fenoterol (400 μg) or ipratropium bromide (80 μg)).

*The six-minute walking test* (6MWT) was performed in accordance with the standard protocol [17]. Prior to and at the end of the test, pulse and oxygen saturation of arterial blood were measured by pulse oximetry using a Nonin 8500 apparatus (USA); dyspnea was assessed on a modified 10-point Borg scale.

### 2.3 Psychiatric Examination

All patients were evaluated by a trained psychiatrist (P.D.S) with two reliable questionnaires – the Hamilton depression rating scale and the Hamilton anxiety depression rating scale. The psychiatrist was unaware of the aim of the study at the time of evaluation and did not have information on Brief-IPQ results.

### 2.4 Illness Perception

was evaluated using a shortened version of the Illness perception questionnaire (Brief IPQ). Psychometric properties of Brief IPQ were tested by E. Broadbent *et al*. in a population of patients with different chronic diseases [18]. It was found to be a reliable and valid tool to assess illness perception in outpatients and large-scale studies. Brief IPQ was validated in Russia by V. Yaltonsky *et al*. [19].

Statistical analysis was carried out using the SPSS Statistics v.17.0 software package and Microsoft Excel. Cluster analysis was carried out using hierarchical clustering; the results were presented in a dendrogram. The chi-square test was used to assess the validity of the differences in qualitative variables in two independent groups. A comparison of the means between the three selected groups was carried out using the Kruskell-Wallace test with Boneferroni's correction. Differences were considered significant at p <0.05;

## 3 RESULTS

One hundred forty-three patients (83%) out of the 172 initially included in the study completed all the questionnaires presented. Their average age was 66.2±7.6 years. Males predominated and made up 107 observations (74.8%). Eighty-seven patients were married at the time of inclusion in the study (67.8%), 57 patients had a regular job (39.8%). Their socioeconomic status and educational level were relatively low; in 102 cases (71.3%), the socioeconomic status was classified as low, and only 69 patients (48.2%) had higher education. The duration of COPD in this sample was relatively short – 5.4±3.2 years. COPD with a rather severe course prevailed in the sample as stage III-IV COPD according to GOLD was established in 95 cases (69%). The severity of dyspnea on the mMRC scale in this sample was relatively moderate; in most patients, the severity of dyspnea scored 2 or 3 points out of 5 (96 observations, 69.5%); severe and very severe dyspnea (4 or 5 points) was discovered only in 23 and 4 observations, respectively (16.8% and 2.9%, respectively).

### 3.1 Cluster Analysis

Using the method of hierarchical clustering, we identified 3 principal clusters of illness perception. Clusters I and II were more prevalent (n=72 and n=58, respectively), while only 13 observations were included in cluster III. Reclustering using 50% of the sample gave similar results with the selection of three main clusters, consisting of 36, 28 and 7 participants.

Significant differences in illness perception between clusters were identified in almost all brief-IPQ items, with the exception of item 2 (how long illness will last) and item 7 (understanding of disease) (Table **1**). Clusters I and II showed opposite illness perception profiles. Сompared with cluster II, patients from cluster I perceived their disease as less affecting (item 1), felt much more control over the illness (item 3), they were more sure that treatment would help (item 4), had much less concern and experienced a smaller emotional impact of the disease (items 6 and 8) (Table **2**). Furthermore, patients from cluster I generally described their disease as less symptomatic (item 5) (p = 0.021), but the difference in this parameter was less prominent than in the rest. Hence cluster I of illness perception was named “disregarding”, and cluster II was named “distressed.”

**Fig. (1):**
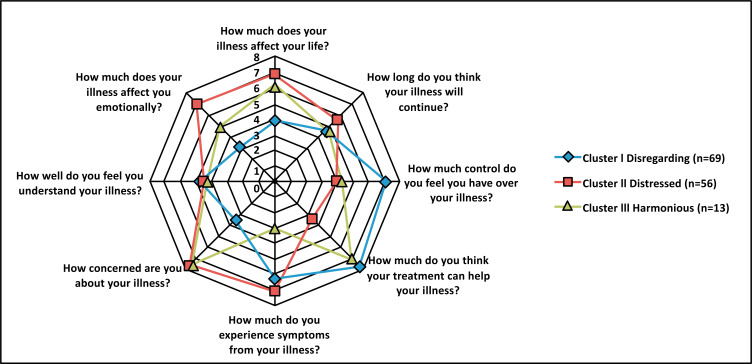
Illness perception profiles of the clusters.

In terms of illness perception, patients from cluster III, in turn, occupied an intermediate position between clusters I and II, showing similarities in some parameters with cluster I, and in other parameters with cluster II (Table **2** and Fig. **1**). Thus, when compared to cluster I, patients in cluster III had the same belief in their ability to control their illness (item 3) and were similarly sure that treatment would help them (item 4). At the same time, patients from cluster III were less optimistic about the influence of the disease on their lives (item 1), had significantly more concerns about COPD (item 6), and were more affected emotionally (item 8). Once we compared cluster III with cluster II, statistically significant differences were also revealed. Patients in сlusters II and III assessed the impact of the disease on their lives in the same manner (item 1) and experienced an equivalent level of concern for COPD (item 6). At the same time, patients from cluster III were significantly more optimistic about having control over the disease (item 3), the effectiveness of treatment (item 4) and also experienced a significantly smaller emotional impact of COPD (item 8). In addition, patients from cluster III experienced fewer COPD symptoms than cluster II patients, as in the case of cluster I (item 5). In this regard, cluster III was designated as “harmonious.”

### 3.2 Differences between Clusters

The clusters identified in this study were characterized by significant differences in a number of socio-demographic and clinical parameters (Table **3**). In total, statistically significant differences were identified for ten parameters, total duration of COPD, smoker status at the time of inclusion in the study, a history of attempts to quit smoking among current smokers at the time of enrollment, number of cigarettes per day, smoking index, the severity of dyspnea according to the Borg test after a 6-minute walk, anxiety, and depression according to the Hamilton questionnaires (mean values ​​for clusters and percentage of patients without depression/with mild anxiety). At the same time, there were no statistically significant differences in such parameters as gender, age, marital status, employment status, educational status, and socioeconomic status. In addition, the objective severity of COPD (FEV1, mMRC, GOLD stage, oxygen saturation after a 6-minute test) also did not differ between clusters.

The duration of COPD was significantly higher in the distressed cluster compared with the disregarding one (Table **4**). Among the distressed patients, there were significantly fewer smokers and fewer attempts to quit smoking in current smokers. Moreover, those distressed patients who smoked at the time of inclusion in the study smoked fewer cigarettes, and their smoking index was significantly lower. An unexpected result was a significantly greater severity of dyspnea after the 6-minute walk test in the distressed cluster, which clearly contrasted with a comparable level of oxygen saturation and a similar FEV1 between them and the disregarding cluster. Finally, according to Hamilton's questionnaires, anxiety and depression were significantly higher in the distressed cluster in comparison with the disregarding one. At the same time, the average Hamilton score for depression in the distressed cluster was 7.9 ± 4.3, which corresponds to mild clinical depression, and the average Hamilton anxiety score was 22 ± 6, which corresponds to moderate anxiety. In the disregarding cluster, there were only 2 patients with moderate anxiety and none with severe anxiety, as well as only 3 patients with mild depression. In turn, in cluster II, there were 46 (almost 80%) patients with moderate and severe anxiety, and depression was diagnosed in 20 patients (34.5%) according to the Hamilton questionnaire.

The harmonious cluster occupied an intermediate position between the other two clusters. These patients did not significantly differ from the disregarding cluster in the duration of COPD, the number of smokers, the severity of dyspnea after a 6-minute test, as well as Hamilton's depression and anxiety scores. At the same time, the harmonious cluster had a slightly different profile in relation to smoking. Current smokers from the cluster were significantly more likely to try to quit smoking in the past, and the harmonious cluster as a whole was characterized by a lower smoker index; in addition, the patients in this cluster had a tendency to smoke fewer cigarettes per day (p = 0.06).

When comparing harmonious and distressed clusters, a significant difference was found in the severity of dyspnea after a 6-minute walk test, as well as in depression and anxiety scores. At the same time, the duration of COPD, as well as all parameters related to smoking, did not show significant differences. None of the patients assigned to cluster III scored enough on anxiety or depression scales to pass the threshold for diagnosis.

## 4 DISCUSSION

The present paper is the first known study that investigates illness perception in COPD patients and is based on brief-IPQ with cluster analysis. In addition, this is the first research where illness perception clusters in COPD were assessed with validated clinical questionnaires used by a qualified psychiatrist.

Three different clusters of disease perception were identified in our sample. The identified clusters did not have statistically significant differences in relation to the severity of the disease or main socio-demographic parameters. In this regard, it can be argued that the differences found between them were rather reflective of the characteristics of the illness perception.

The first of the identified clusters - the “disregarding” - was defined by an underestimation of the impact of the disease on a patient’s life (item 1), a patient’s belief in their ability to control their disease and recover with the help of drugs (items 3 and 4), along with minimization of concerns and emotional experiences associated with the disease (items 6 and 8). This cluster was characterized by a higher percentage of smokers, a higher smoker index, along with low scores on the Hamilton Anxiety and depression scales. Patients of this cluster were the most numerous, which is consistent with the relevant literature arguing that COPD patients tend to underestimate the threat from COPD and demonstrate low adherence to medical recommendations [20].

Patients of the second cluster - the “distressed” - had an opposite perception of COPD. They exhibited disbelief in their ability to control their disease (item 3) and doubted the efficacy of treatment (item 4). The distressed patients also had much more concern and a stronger emotional response to this illness (items 6 and 8). The medical behavior of this group was formally healthier than among the disregarding; there were significantly more non-smokers, and almost all of the current smokers in this cluster had made attempts to quit smoking in the past. The smoking index and the number of cigarettes per day were also lower. According to other research on this topic, anxiety in patients with COPD is a factor that may prompt them to keep smoking [21]; however, in our study, the group with the highest severity of anxiety was the least inclined to smoking. This effect may be mediated by their perception of illness rather than the anxiety itself.

The most interesting result in the distressed cluster was a significantly higher mean score for subjective dyspnea on the modified Borg scale after the 6-minute walk test. This increase cannot be explained by differences in respiratory status, as there was no significant difference with other clusters in parameters such as FEV1 and oxygen saturation after a 6-minute walk test. Apparently, the overestimation of the severity of dyspnea is associated with pessimistic illness perception and a high level of anxiety. This hypothesis is confirmed by contemporary medical literature. In particular, N. Livermore *et al*. demonstrated a greater severity of dyspnea according to the modified Borg scale after exercise in patients with comorbid anxiety and COPD compared with COPD patients without anxiety [22].

The small size of the third cluster – the “harmonious” – can be explained by the peculiarities of the sample of patients in this study, such as the prevalence of smoking patients, low socioeconomic status, and the relatively short duration of COPD. Patients of this cluster were characterized by a combination of a belief that treatment could help them (item 4) and a lack of confidence in their ability to control their disease (item 3). The patients in this cluster reported prominent concerns about the disease (item 6) but experienced a smaller emotional impact (item 8). The results obtained strongly indicate that the harmonious cluster, which formally occupies an intermediate position between clusters I and II, is not transitional between them; on the contrary, it represents a special form of perception of the disease (Fig. **1**). This form of perception of COPD seems to be the most adaptive. On the one hand, patients show a greater tendency to adjust their lifestyle as they smoke less than the disregarding and make more attempts to quit smoking. On the other hand, contrary to the distressed cluster, they have no clinically apparent anxiety or depression and perceive less dyspnea after physical activity.

The most interesting result in relation to the harmonious cluster is that the patients belonging to it seem to be experiencing fewer COPD symptoms (question 5 of the brief IPQ questionnaire) in comparison with other clusters. The design of this study does not allow for an unambiguous interpretation of this phenomenon. Since there are no differences in the clinical severity of COPD between clusters, the lower declared severity of COPD in patients with harmonious type needs to be clarified in further studies.

In the available literature, two studies were found that were dedicated to the COPD perception using cluster analysis [9, 23]. Both studies were carried out using a modified IPQ-R, rather than a short version of the questionnaire, but their results are quite comparable to those of our research due to a high level of similarity between the questionnaires (with the exception of the cyclical course of the disease subscale, which is not reflected in the brief version).

According to the formal inclusion criteria, the patients of our study were more similar to those included in Lopes’s study (outpatients who did not experience exacerbations of COPD for several months). However, Lopes *et al*. identified only two clusters - the “distressed” and the “coping.” There were no analogs for the disregarding cluster. Apparently, this difference is associated with the peculiarities of the formed samples and national trends in smoking. In our study, more than half of the patients continued to smoke despite a fairly long course of COPD, while only a few of the patients included in the Lopes study were smokers (4 patients, 2.6%). Such a striking difference in smoking profiles may reflect both national differences in attitudes towards smoking (as of 2015, 39% of the population smoke in the Russian Federation, and 10% in Brazil) [24], as well as a different perception of COPD by the Russian patients.

A comparison with Harrison's results can be made with considerable caution as the cited study included patients who recently had an exacerbation of COPD, which contradicts the inclusion criteria for our study [23]. However, the authors received a clustering broadly comparable to ours, along with patients in control (similar to harmonious) and the distressed, and a group of disengaged patients was identified. The latter group was characterized by the presence of a weak emotional reaction to the disease, the description of a smaller number of negative consequences and weaker conviction of the chronic nature of the disease. At the same time, the patients of this group were characterized by their disbelief in their ability to independently control their disease, which can be explained by the inclusion of patients who had a recent exacerbation.

The three types of illness perception that were identified with cluster analysis are partially comparable to the categories of anxious/depressive^1^, hyponosognosic and harmonious types that were identified earlier with the use of a clinical (phenomenological) method [15]. This clinical concept is conditionally comparable to the Yerkes-Dodson law, according to which optimal adaptation to the situation (in this case, to chronic pulmonary disease) is achieved at a certain range of stress and involvement in the situation. Both excessive involvement in a situation with excessive stress levels (hypernosognosia) and decreased perception of stress lead to maladaptive behavior towards this illness. In the present study, we confirmed the existence of the described illness perception types in COPD patients with the assistance of statistical tools.

The revealed differences between the clusters confirm the need for routine assessment of illness perception in patients with COPD. In addition, it can be assumed that patients with a different profile of illness perception need different rehabilitation and support programs. Thus, the disregarding patients are more in need of programs aimed at increasing awareness of the nature of their disease and smoking reduction. At the same time, the distressed patients need psychotherapeutic assistance aimed at correcting anxiety, depression, and a catastrophic illness perception; in the presence of clinically delineated mental disorders, they may require psychopharmacotherapy.

The clusters identified in this paper deserve further examination in prospective studies. Such studies could be observational (to let us assess how the profile of the illness perception affects its course) or interventional (where we can evaluate how the effectiveness of rehabilitation and psychotherapeutic programs correct a patient’s perception of their disease).

## 5 LIMITATION OF THE STUDY

The main methodological limitation of this study is the small size of the harmonious cluster.

Also, the high prevalence of smokers in this sample makes it difficult to generalize the results obtained on non-smoking COPD patients.

The distressed patients in our sample had predominantly moderate anxiety and mild depression. Therefore, it is unclear to what extent clinically delineated severe anxiety or depression, as well as other mental disorders, affect the perception of illness.

The clusters described in the article were identified using an atheoretical statistical tool (cluster analysis); however, the qualification of the patient's illness perception was based on the subjective judgment of the authors, who did not examine the patients directly. In this regard, there is a need for research where the filling of the scales would be accompanied by clinical interviews aimed at clarifying the psychological profiles of patients.

The cross-sectional design of the study does not allow drawing conclusions about the causal relationship between the type of illness perception, socio-demographic, and clinical variables.

Besides, due to the cross-sectional nature of the study, it is impossible to understand to what extent the type of illness perception affects the prognosis and course of COPD.

Another significant limitation is the lack of data on the personality traits of patients with COPD. This data may be more reliable than illness perception profiles and may serve as their predictors.

## CONCLUSION

In this study, we used the cluster analysis method and identified 3 types of illness perception, disregarding, harmonious, and distressed. The results obtained confirm the data of previous studies on the need for routine assessment of illness perception in patients with COPD.

## Figures and Tables

**Table 1:** Illness perception comparison in different clusters.

-	**All Patients (n=143)**	**Cluster I Disregarding (n=72)**	**Cluster II Distressed (n=58)**	**Cluster III Harmonious (n=13)**	**P-value**
How much does your illness affect your life? (item 1)	5.4±0.19	3.9±1.2	6.9±0.2	6.1±0.4	<0.001
How long do you think your illness will last? (item 2)	5.0±1.2	4.7±0.2	5.6±0.2	4.8±0.6	0.857
How much control do you feel you have over your illness? (item 3)	5.6±0.2	7.1±0.2	4.0±0.2	4.3±0.5	<0.001
How much do you think your treatment can help your illness? (item 4)	5.9±0.2	7.7±0.1	3.2±0.2	7.0±0.3	<0.001
How much do you experience symptoms from your illness? (item 5)	6.2±0.1	6.2±0.2	7.0±0.2	3.0±0.3	<0.001
How concerned are you about your illness? (item 6)	5.6±0.2	3.5±0.1	7.7±0.2	7.5±0.4	<0.001
How well do you feel you understand your illness? (item 7)	4.6±0.2	4.7±0.3	4.5±0.3	4.3±0.4	0.729
How much does your illness affect you emotionally? (item 8)	4.9±0.2	3.1±0.1	7.0±0.2	5.0±0.5	<0.001

**Table 2:** Pairwise comparison of statistically significant differences of brief IPQ-R questions between clusters.

-	**Disregarding *vs.* Harmonious**	**Disregarding *vs.* Distressed**	**Distressed *vs.* Harmonious**
How much does your illness affect your life? (item 1)	0.02*	<0.001*	0.961
How much control do you feel you have over your illness? (item 3)	1.0	<0.001*	<0.001*
How much do you think your treatment can help your illness? (item 4)	0.831	<0.001*	<0.001*
How much do you experience symptoms from your illness? (item 5)	<0.001*	0.021*	<0.001*
How concerned are you about your illness? (item 6)	<0.001*	<0.001*	1.0
How much does your illness affect you emotionally? (item 8)	0.021*	<0.001*	0.01*

**Table 3:** Patient characteristics (n=138) and between-cluster differences on all measured variables.

-	**All Patients (n=143)**	**Cluster I Disregarding (n=72)**	**Cluster II Distressed (n=58)**	**Cluster III Harmonious (n=13)**	**P-value**
Gender (% of males)	107 (74.8%)	59 (81.9%)	38 (65.5%)	10 (76.9%)	0.065
Age, years	66±7.5	65±8.1	67±6.8	68±6.9	0.167
Marital status (% of married particpants)	87 (60.8%)	41 (56.9%)	39 (67.2%)	7 (53.8%)	0.134
Employment status (% of current workers)	57 (39.8%)	27 (37.5%)	23 (39.6%)	7 (53.8%)	0.61
Socioeconomic status (low)	102 (71.3%)	53 (73.6%)	43 (74%)	6 (46%)	0.072
Education (% of highly educated particpants)	69 (48.2%)	34 (47.2%)	28 (50%)	7 (53.8%)	0.83
Duration of COPD years	5.4±3.2	4.54±2.3	6.5±4	5±1.7	0.026*
Predicted FEV1%	51.5±16.5	50.7±6.2	51.6±16.2	55.8±16.5	0.562
mMRC	2.75**±**0.96	2.8±1	2.8**±**0.83	2.5±0.96	0.087
GOLD stage	3±1.2	3±1.2	3±1.2	2.5±1.3	0.213
COPD exacerbation per year	1.95±0.97	1.93±0.9	2.05±0.9	1.62±0.8	0.297
Current smoker (%)	94 (65.7%)	54 (75%)	30 (51.7%)	10 (76.9%)	0.01*
History of attempts to quit smoking (% of those who tried to quit among current smokers)	68 (72.3%)	30 (55.5%)	29 (96.7%)	9 (90%)	<0.001*
Number of cigarettes per day	23±9.1	26±9.6	18.9±6.6	21.6±9.6	<0.001*
Smoker index (pack*years)	50.5±23.2	57.2±25.2	41.6±15.7	50.1±28.3	0.001*
Saturation after 6MWT	94±2.9	94±2.9	93.8±3	95.3±1.7	0.317
Borg dyspnea after 6MWT	1.7±1.2	1.3±0.98	2.4±1.7	0.69±0.5	<0.001*
HARS	14.6±8	9.7±4.4	22±6	8.85±4.5	<0.001*
Number of patients with moderate/severe anxiety	48 (33.6%)	2 (2.7%)	46 (79%)	0 (0%)	<0.001*
HDRS	5.5±3.7	3.9±2	7.9±4.3	3±2	<0.001*
Number of depressed patients (%)	23 (16%)	3 (4%)	20 (34.5%)	0 (0%)	<0.001*

**Table 4:** Pairwise comparison of statistically significant p-values (after Bonferroni correction).

-	**Disregarding *vs.* Harmonious**	**Disregarding *vs.* Distressed**	**Distressed *vs.* Harmonious**
Duration of COPD Years	1.0	0.02	1.0
Current Smoker (%)	1.0	0.01	0.315
History of attempts to quit smoking (% of those who tried to quit)	<0.001*	<0.001*	1.0
Number of Cigarettes per Day	0.06	<0.001*	0.83
Smoker Index (pack*years)	0.05	0.001*	0.757
Borg Dyspnea after 6MWT	0.78	<0.001*	0.003
HARS	1.0	<0.001*	<0.001*
Number of Patients with moderate/severe anxiety	1.0	<0.001*	<0.001*
HDRS	0.64	<0.001*	<0.001*
Number of Depressed Patients (%)	1.0	<0.001*	<0.001*

## Data Availability

The data and materials used to support the findings of this study are available from the corresponding author [D. P.], upon reasonable request.

## Footnotes

1 Label “distressed” is much more appropriate for further studies because not every distressed patient actually has anxiety or depression
